# High Aspect Ratio Polymer Nanocarriers for Gene Delivery and Expression in Plants

**DOI:** 10.1021/acs.nanolett.4c04704

**Published:** 2024-12-31

**Authors:** Yilin Zhang, Jinwoo Shin, Hui Sun, Hsin-Fang Chang, Michael R. Martinez, Lydia A. Perkins, Jiajun Yan, Yunteng Cao, Hairong Wang, Juan Pablo Giraldo, Krzysztof Matyjaszewski, Jen Sheen, Robert D. Tilton, Benedetto Marelli, Gregory V. Lowry

**Affiliations:** ^†^Department of Civil and Environmental Engineering, ^‡^Center for Environmental Implications of Nano Technology (CEINT), ^§^Department of Chemistry, ^∥^Department of Chemical Engineering, ^⊥^Department of Biomedical Engineering, and ^#^Department of Biological Sciences, Carnegie Mellon University, Pittsburgh, Pennsylvania 15213, United States; g Department of Molecular Biology and Centre for Computational and Integrative Biology, Massachusetts General Hospital, and Department of Genetics, Harvard Medical School, Boston, Massachusetts 02114, United States; h Department of Civil and Environmental Engineering, Massachusetts Institute of Technology, Cambridge, Massachusetts 02139, United States; i Department of Botany and Plant Sciences, University of California, Riverside, California 92521, United States

**Keywords:** gene delivery, sustainable agriculture, nanocarrier, bottlebrush polymer, foliar application

## Abstract

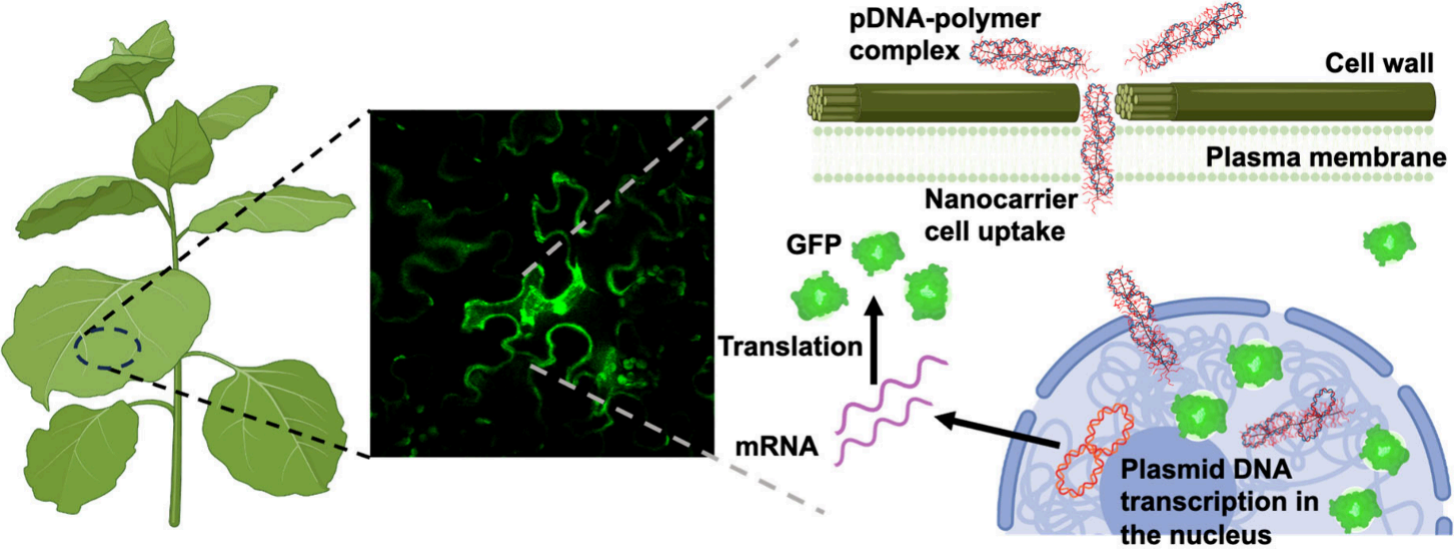

Plant genetic engineering methods are critical for food security and biofuel production and to enable molecular farming. Here, we elucidated how polymeric high aspect ratio nanocarriers can enable DNA delivery to *Nicotiana benthamiana* plants and transient expression. We demonstrated that a nanocarrier with 20 nm width, 80 nm length, and a polymer-to-DNA ratio of N/P = 3.0 afforded the most efficient DNA delivery and expression among the parameter space investigated. Additionally, we showed that polymer–DNA complexes with a moderate positive charge of ∼14 mV favored penetration through the cell wall and membranes with the assistance of cell wall degrading enzymes. Together, these results establish a narrow window of aspect ratios and charges of the nanocarrier–DNA complex that enables DNA delivery to plants using polymeric nanocarriers. This fundamental nanocarrier structure–function relationship informs the design of soft-material nanocarriers for nucleic acid delivery in plant cells to facilitate a wide range of plant biotechnology applications.

The ever-growing global population, a shortage in farmland, and climate change impacts on crop production require new technologies that sustainably fulfill the growing demands for food, energy, medicine, and materials.^1−4^ Plant genetic engineering can help tackle these challenges by enhancing desired plant functions, including higher yields, faster growth, resistance to diseases and environmental stressors, and the opportunity to enable plant-based biomanufacturing.^5−7^ However, current plant genetic transformation technologies have limitations in host range and scalability.^8,9^ The most common *Agrobacterium*-mediated transformation suffers from the risk of mutagenesis^10^ and limited host range,^10^ which restrains their applications in diverse crops.^10^ The other widely used plant transformation technology, gene gun bombardment, works for a wider range of plants but still suffers from random mutation and tissue damage that can result in low transformation efficiencies.^11,12^ The regeneration of plants from genetically modified plant cells is also a long and intensive process.^13^ The expensive, specialized devices and complex protocols also limit their application in high-throughput conditions, such as agriculture.^8,11^ An alternative strategy that can effectively deliver nucleic acids into plants is needed to address these limitations.

Nanomaterials are promising tools for plant genetic engineering.^14,15^ A variety of nanocarriers have been developed to carry nucleic acids through plant barriers, including cuticles, cell walls, and cell membranes.^16^ DNA nanostrings with a 5 nm width and 320 nm length can penetrate cell walls and deliver gene silencing siRNA into *Nicotiana benthamiana* (*Nb*) plant leaf cells.^17^ Gold nanoclusters and gold nanorods with widths below 20 nm can also deliver siRNA into *Nb* plants.^18,19^ Soft micelles composed of cell-penetrating peptides (∼100 nm diameter) have enabled targeted nucleic acid delivery into plant mitochondria, chloroplasts, and cell nuclei.^5,20,21^ Foliar applied layered double hydroxide clay nanosheets delivered double-stranded RNA to cowpea plants, providing at least 20 days’ protection against pathogens.^22^ Polyethylenimine-functionalized carbon dots with ∼4 nm diameter have delivered functional nucleic acid into wheat plants.^23^ The majority of nucleic acid delivery studies have focused on RNA delivery, which is much smaller than plasmid DNA, and structure–function relationships are still needed for the efficient nanocarrier design for nucleic acid delivery in plants.

High aspect ratio nanomaterials are promising tools for efficient DNA delivery and plant genetic engineering.^10,24^ Rigid single-walled carbon nanotubes (SWCNTs) functionalized with polyethylenimine delivered plasmid DNA and siRNA to *Nb* plant cell nuclei for expression.^10,25,26^ Chitosan or biorecognition peptide-functionalized SWCNTs can also achieve targeted plasmid DNA delivery and expression in *Arabidopsis thaliana* and arugula chloroplasts, with potential for chloroplast bioengineering.^11,27^ While a high aspect ratio has been proposed to promote nucleic acid delivery in plants,^10^ the aspect ratio of current nanocarriers used for this purpose, such as SWCNTs, cannot be easily controlled and the direct impact of nanomaterial aspect ratio on DNA delivery and expression in plants remains unclear.

Bottlebrush polymers are densely grafted copolymers with a large aspect ratio and elongated structure in solution.^28−32^ They can efficiently enter cells, depending on their dimensions and the aspect ratio.^33−36^ Bottlebrush polymer and star polymer nanocarriers with highly controllable aspect ratios and chemical compositions are a promising platform for DNA delivery in cells^37−40^ and to examine the impact of nanocarrier properties on gene delivery and expression in plants. Here, we synthesized a library of different aspect ratio cationic bottlebrush polymer nanocarriers. The nanocarrier charge and morphology were controlled by mixing with plasmid DNA at different nitrogen to phosphate molar ratios (N/P = 0.5–3.0) to investigate the effect of nanocarrier aspect ratio and charge on their DNA delivery efficiency in plants. The conformation of different nanocarrier–DNA complexes was assessed by atomic force microscopy (AFM) to determine the ideal morphologies for gene delivery.

Cationic polymer nanocarriers with various aspect ratios, including 21-armed star polymer (SP), 320-armed short polymer bottlebrush (SPBB), and 1600-armed long polymer bottlebrush (LPBB), with 50 degrees of polymerization (DP) poly(2-(dimethyl amino)ethyl methacrylate) (PDMAEMA) side chains were synthesized by atom transfer radical polymerization (Figure 1a).

**Figure 1:**
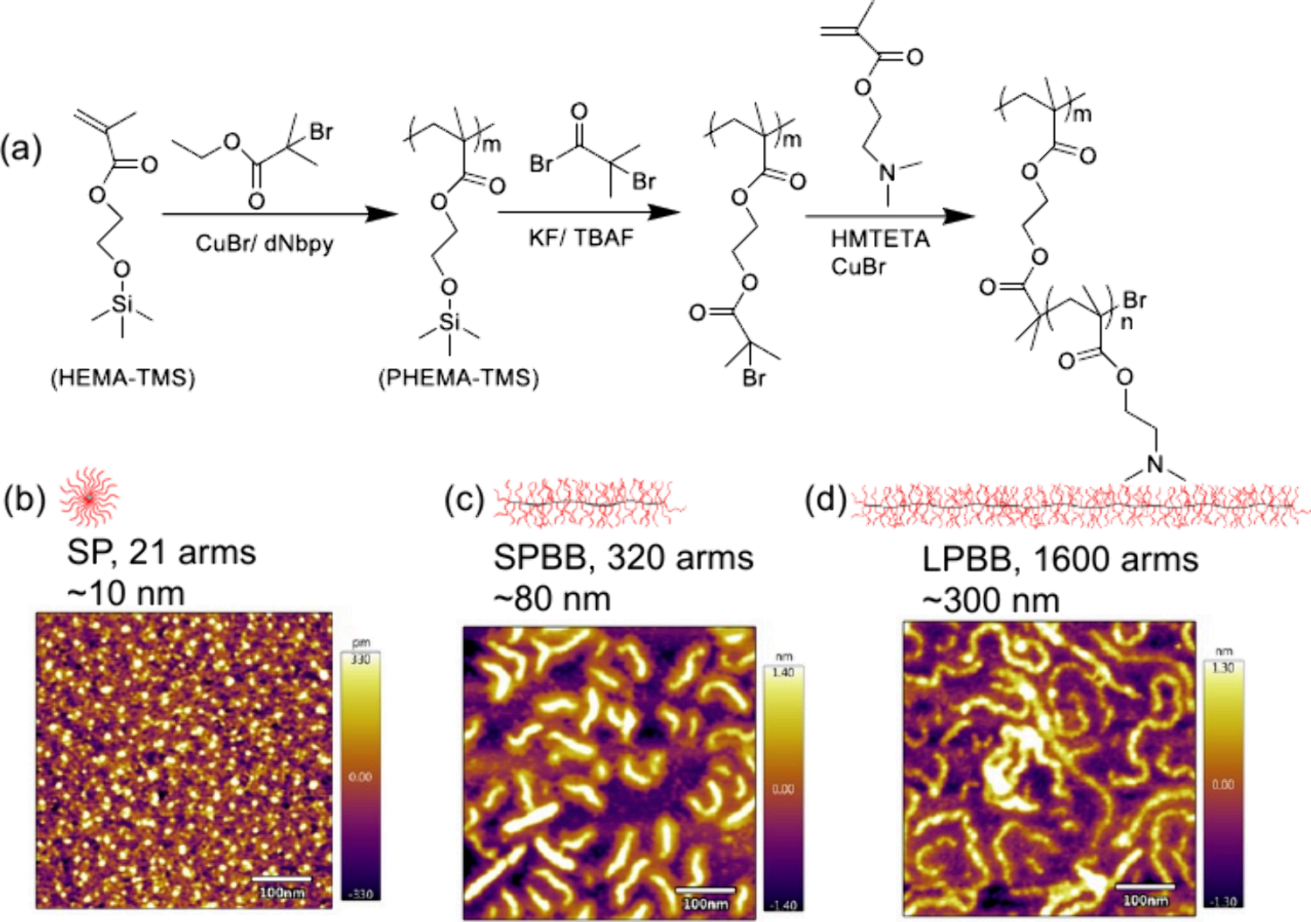
Synthesis and characterization of cationic polymer nanocarriers. (a) Synthesis procedure for cationic bottlebrush polymer nanocarriers. Height profiles of (b) SP, (c) SPBB, and (d) LPBB assessed by atomic force microscopy.

Different aspect ratios of polymer nanocarriers were verified by AFM (Figure 1b–d). The spherical SPs are round shaped with an ∼10 nm diameter (Figure 1b). High aspect ratio bottlebrush polymers are generally rod shaped for the ∼80 nm SPBB (Figure 1c) and have a less linear “wormlike structure” for the ∼300 nm LPBB (Figure 1d).

The tertiary amine in nanocarrier side chains possess a strong positive charge, which enables their complexation with anionic nucleic acids through electrostatic attraction.^37,41^ The plasmid DNA used in this study is *UBQ10-Dof1-sGFP(S65)-NOS*, which contains a nuclear localization signal (Dof1a) to encode GFP that can accumulate in the plant cell nucleus (Figure S3).^42^ The expressed GFP can light up the cell nucleus of infected dicot *Nb* plant protoplasts, validating their effectiveness (Figure S4). Before DNA loading, all nanocarriers are highly cationic with an apparent zeta potential of 24.7 ± 4.9, 20.3 ± 0.3, and 32.9 ± 0.8 mV for SP, SPBB, and LPBB, respectively (Figure S2d, Table S2). The charge of the polymer–DNA complex was adjusted by varying the molar ratio of amines (N) in the polymer nanocarriers to phosphates (P) in nucleic acid backbone (N/P ratio). N/P ratios of 0.5, 1.5, and 3.0 were used in this study, which are common ratios for nucleic acid delivery in animal and plant cells.^8,16,37^ N/P = 0.5 results in excess DNA and a negative apparent zeta potential for the formed polymer–DNA complexes.^16,37^ The ζ-potentials of SP, SPBB, and LPBB were −9.9 ± 0.8 mV, −9.4 ± 1.8 mV, and −7.9 ± 1.1 mV, respectively at N/P = 0.5 (Figure S2d, Table S2). N/P = 1.5 has more polycations, which result in a slight positive charge (7.4 to 19.6 mV) of the complexes (Figure S2d, Table S2), significantly higher than N/P = 0.5 (*p* < 0.05). N/P = 3.0 results in complexes with moderately positive ζ-potentials of 18.1 ± 2.3 mV, 14.2 ± 2.3 mV, and 19.5 ± 2.7 mV for SP, SPBB, and LPBB (Figure S2d, Table S2). The net charge increased for SPBB and LPBB as the N/P ratio increased from 1.5 to 3.0 (*p* < 0.05). Hydrodynamic diameters of polymer–DNA complexes were also analyzed by dynamic light scattering (DLS). The overall size of most polymer–DNA complexes did not change significantly (Figure S2e, Table S2).^37^

Complexation of different aspect ratio nanocarriers with DNA at the same N/P ratio (N/P = 3.0) led to drastic conformational changes for SP and LPBB, but not for the SPBB (Figure 2a–c). Due to the large molecular weight and volume of plasmid DNA molecules, as well as the nonrigid nature of polymer nanocarriers, the DNA complexation can result in bending, intermolecular aggregation, and intramolecular aggregation of high aspect ratio nanocarriers, which change the morphology of nanocarrier–DNA complexes. DNA complexation with SP resulted in spherical nanoparticles with an average diameter of 147 ± 36 nm and a height of 13.4 ± 5.7 nm (Figure 2a,g,h), much larger and thicker than the pristine SP (Figure 1b). The size of the plasmid DNA (5451 bp) is much larger than that of the SP according to their AFM height profile (Figure S5). Instead of DNA loading onto this nanocarrier, it is more likely that multiple smaller SPs assembled around larger DNA molecules to form spherical nanoparticles. This is consistent with previous studies, where small cationic peptides formed spherical structures with 123 ± 13 nm diameter after being complexed with plasmid DNA.^8^ The longer LPBB formed 126 ± 19.2 nm wide and 9.0 ± 2.4 nm high oval-shaped aggregates after being complexed with DNA (Figure 2b,g,h). The 50 DP PDMAEMA cannot provide sufficient repulsion between its arms to maintain the worm-like conformation of the LPBB after being complexed with DNA, resulting in aggregation and an increased width. In contrast to LPBB, the shorter SPBB mostly preserved its rod shape after being loaded with DNA at N/P = 3.0 (Figure 2c), with a width of 40.1 ± 5.8 nm and a height of 3.6 ± 0.6 nm (Figure 2g,h), and is much smaller than the size of either the SP or the LPBB after DNA loading.

**Figure 2:**
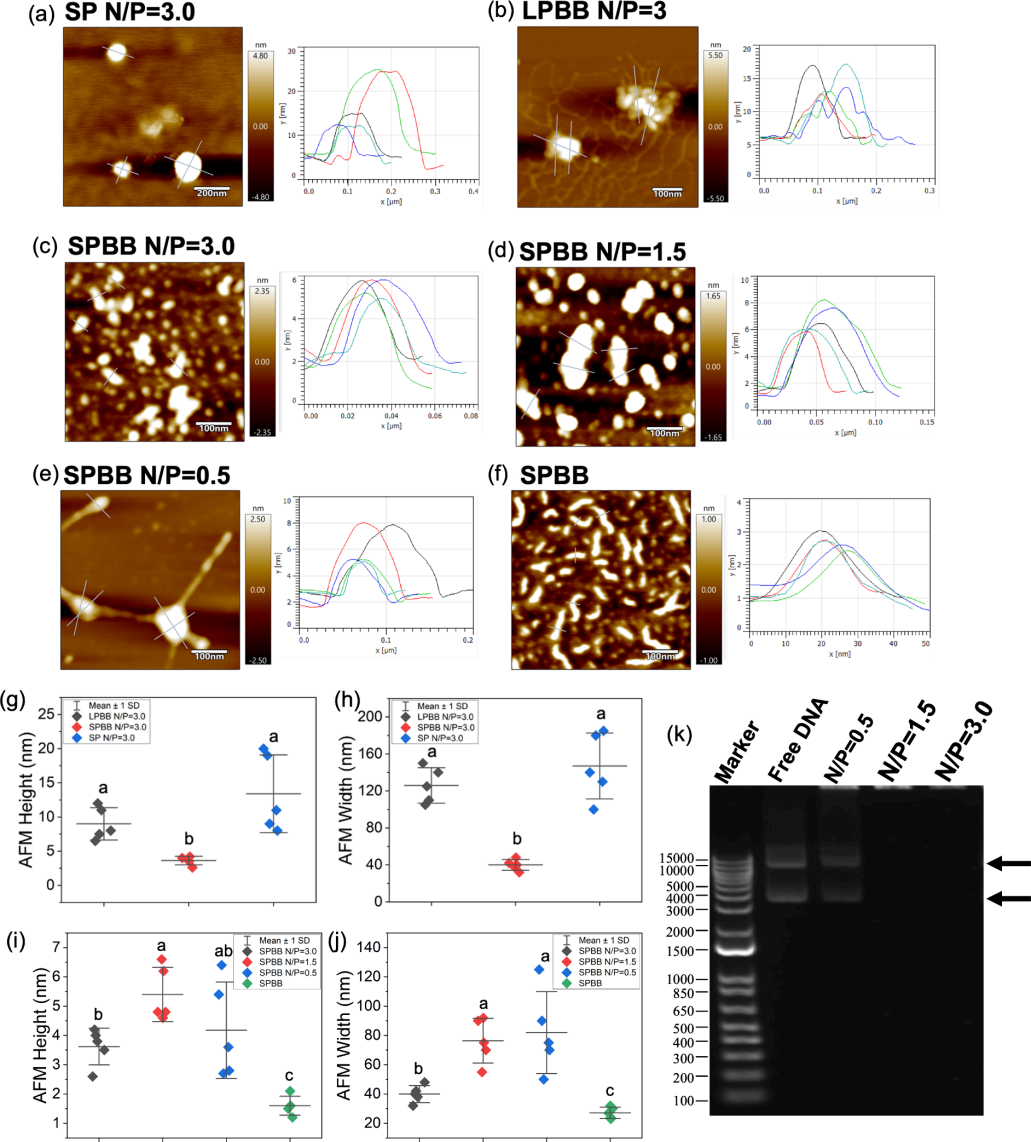
Complexation of DNA with cationic polymer nanocarriers. Height profiles of (a) SP, (b) LPBB, and (c) SPBB complexed with DNA at N/P = 3.0, SPBB–DNA complexes at (d) N/P = 1.5, and (e) N/P = 0.5, and (f) unloaded SPBB assessed by AFM. (g) AFM height and (h) width of SP, SPBB, and LPBB complexed with plasmid DNA at N/P 3.0 and (i) AFM height and (j) width of unloaded and DNA-loaded SPBB at N/P 0.5, 1.5, and 3.0. Error bars represent standard deviation (*n* = 5). ANOVA testing followed by Fisher’s LSD testing was used for multiple comparisons, *p* ≤ 0.05. (k) DNA loading into SPBB quantified by agarose gel electrophoresis of plasmid DNA complexed with SPBB at 0.5, 1.5, and 3.0 N/P ratios. Black arrows indicate supercoiled (bottom) and circular (top) plasmid DNA bands.

The N/P ratio also affected the conformation of the polymer–DNA complexes. The pristine SPBB had a width of 27.2 ± 3.8 nm and height of 1.6 ± 0.3 nm (Figure 2i,j). DNA loading significantly increased the heights and widths of the SPBB at all N/P ratios tested (Figure 2i,j). Loading of excess DNA at N/P = 0.5 created interconnected spherical complexes (Figure 2e) with a width of 82.0 ± 27.9 nm and height of 4.2 ± 1.6 nm (Figure 2i,j). Loading the SPBB with less DNA led to lower height and width of these polymer–DNA complexes, as both the N/P = 1.5 and 3.0 polymer–DNA complexes still have a rod-shaped morphology (Figure 2c,d) with heights of 5.4 ± 0.9 nm and 3.6 ± 0.6 nm (Figure 2i) and widths of 48.7 ± 9.2 nm and 40.1 ± 5.8 nm (Figure 2j), respectively. Nanoparticle uptake into plant cells has been shown to be controlled by the size of their smallest dimension.^11^ The relatively smaller width and height of the N/P = 3.0 SPBB polymer–DNA complex could favor its uptake into plant cells, as later discussed in the DNA uptake study (Figure 3). The gel electrophoresis showed no free DNA moving into the agarose gel at N/P ratios of 1.5 and 3.0 for SPBB, confirming 100% DNA loading into the nanocarriers (Figure 2k). Unbound free DNA bands were observed for an N/P ratio of 0.5 (Figure 2k), suggesting incomplete DNA loading at the lowest N/P ratio. Complete DNA loading into SP and LPBB at N/P = 3.0 is also confirmed by gel electrophoresis (Figure S6).

**Figure 3:**
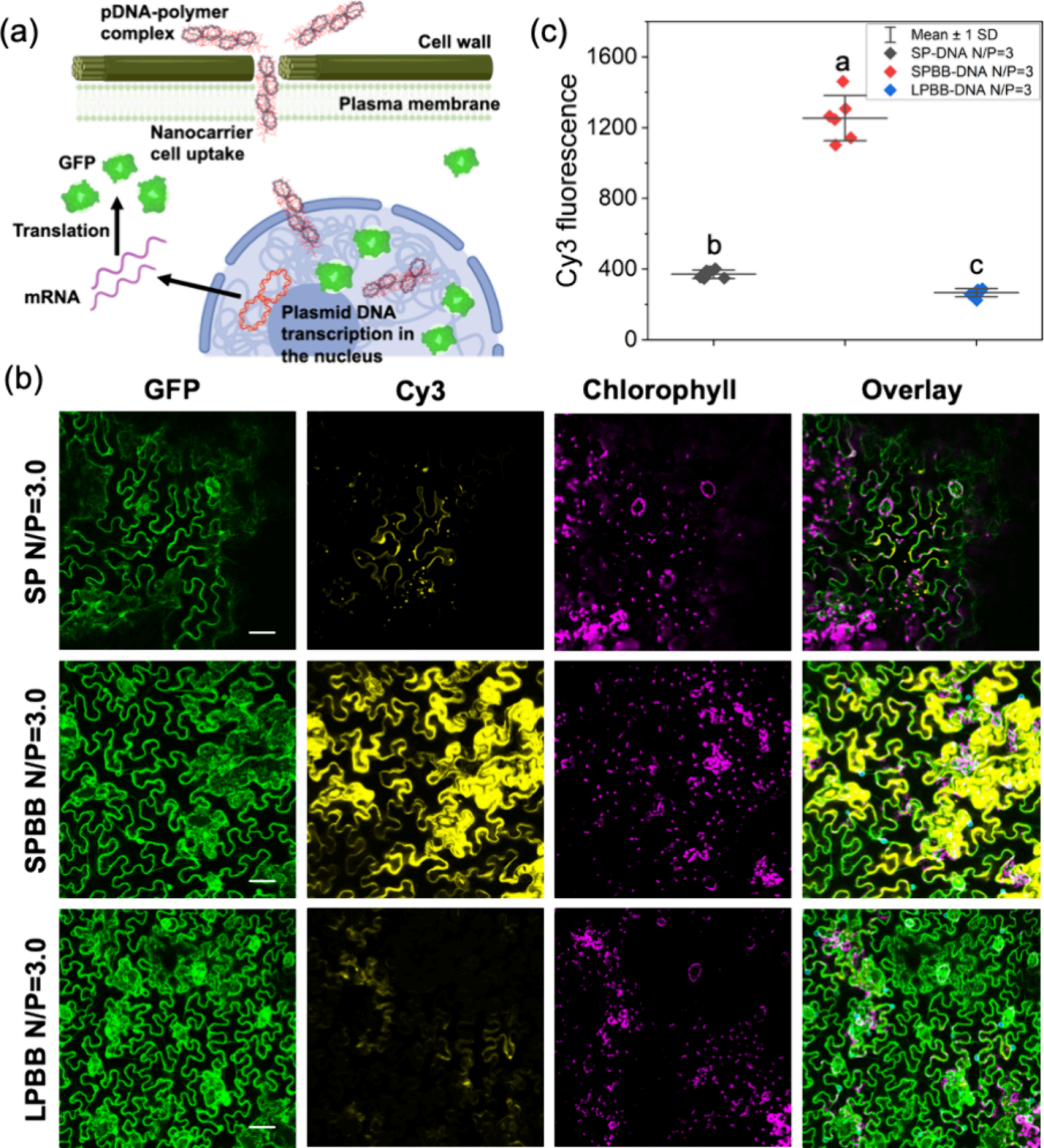
DNA delivery into a transgenic *Nb* plant that constitutively expresses GFP in the cell cytoplasm by nanocarriers with different aspect ratios, including SP, SPBB, and LPBB after foliar application. (a) Schematic illustration of DNA–polymer complex uptake and translocation into plant cell cytoplasm and subsequent DNA expression. (b) DNA–polymer complex internalization into plants after foliar application of Cy-3-labeled DNA loaded to SP, SPBB, and LPBB at N/P = 3.0. (c) DNA uptake efficiency quantified by the Cy3 fluorescence intensity analysis of *Nb* leaf confocal images. Scale bars, 50 μm. Error bars represent standard deviation (*n* = 6). ANOVA testing followed by Fisher’s LSD testing was used for multiple comparisons, *p* ≤ 0.05. *Nb* plants are used in this study due to their high transfection efficiency and strong protein expression, attributed to their weakened mutation defense system compared to other plants.

To examine the effect of nanocarrier aspect ratio on the ability to deliver DNA into plant cells, the different aspect ratio nanocarriers were loaded with Cy3-labeled DNA (Cy3-DNA) at N/P = 3.0, and their distributions were measured with confocal laser scanning microscopy (Figure 3). The N/P = 3.0 ratio was chosen due to the relatively high net charge and small hydrodynamic diameters (Figure S2d,e).^18,19,43^ A transgenic *Nb* plant that constitutively expresses GFP in the cell cytoplasm was used to examine the internalization of Cy3-DNA into leaf epidermal cells by their colocalization with GFP in the cytoplasm. A solution of 0.03% (wt/vol) cellulase and 0.008% (wt/vol) macerozyme was infiltrated to the leaves 24 h before polymer–DNA inoculation to improve cell wall permeability and promote DNA uptake.^42^ After preparation of the polymer–DNA complexes, the suspensions were infiltrated on the abaxial side of the youngest mature leaves of 6-week-old *Nb* plants with a needleless syringe. Significant Cy3-DNA internalization into leaf cells after foliar infiltration was observed in confocal images of the SPBB–DNA complexes as a strong Cy3 fluorescence intensity of 1012 ± 87 in the same focal plane as the intracellular GFP (Figure 3b,c). The SP–DNA and LPBB–DNA complexes had Cy3 fluorescence intensities of 371 ± 24 and 267 ± 23, respectively (Figure 3b,c), weaker than for the SPBB–DNA. These results suggest the DNA delivery efficiencies of SP and LPBB are lower than that of SPBB, likely due to the larger size of both the SP–DNA and LPBB–DNA complexes compared to the SPBB–DNA (Figure 2g,h). No significant DNA uptake into the plant cells is observed without enzyme pretreatment (Figure S7a), suggesting that the cell wall is a major barrier that limits DNA delivery by polymer nanocarriers. Previously developed high aspect ratio nucleic acid nanocarriers are based on stiff SWCNTs that cannot easily deform.^11^ Complexation of DNA with SWCNTs did not result in a significant morphology change. Herein, we elucidated how complexation of nucleic acids with soft nanocarriers possessing a high aspect ratio impacts their DNA delivery efficiency. For soft material based nanocarriers, preserving the overall small widths upon being complexed with nucleic acids is a critical factor for achieving efficient DNA delivery.

Apart from the initial nanocarrier aspect ratios, different N/P ratios also result in different nanocarrier charges and morphologies (Figure S2d, Figure 2i,j) that affect their ability to penetrate through plant cell walls and membranes.^11,44,45^ The effect of N/P ratios on the DNA delivery efficiency in plants was examined by preparing the SPBB–Cy3-DNA complexes at N/P ratios = 0.5, 1.5, and 3.0 to explore the optimal N/P ratio for DNA delivery. The SPBB–Cy3-DNA complexes along with free Cy3-DNA were applied to plant leaves through foliar infiltration, as described above. When the Cy3-DNA was applied to plants without nanocarriers, the Cy3 signal was not internalized into plant cells (Figure 4d,e). This is consistent with previous work, where free DNA cannot be taken up by plant cells.^10,27^ When Cy3-DNA is complexed with the SPBB, a strong and focused signal of Cy3-DNA appears on leaf epidermal cells at N/P = 3.0 (Figure 4a), suggesting effective DNA delivery into *Nb* plant epidermal cells. This strong DNA uptake is also found in mesophyll cells (Figure S7b). The N/P = 0.5 and 1.5 SPBB–Cy3-DNA complexes did not facilitate significant DNA uptake into plant cells, with much lower Cy3 fluorescence intensity than N/P = 3.0 (Figure 4b,c,e). These results indicate that the N/P = 3.0 polymer–DNA complex with moderate positive charge (14.2 ± 2.3 mV) and small width (Figure S2d, Figure 2i,j) is the optimal ratio for DNA delivery into plant cells among all the N/P ratios tested. It is worth noting that incomplete DNA loading at N/P = 0.5 could also contribute to reduced DNA delivery, as free DNA exhibits low cellular uptake efficiency. Previous studies indicated that chitosan-functionalized SWCNTs–DNA complexes need to possess a net charge above ∼20 mV and a small width (<20 nm) to penetrate through cell membranes for DNA delivery into desired plant cell organelles.^46^ Our study suggests that soft polymeric nanoparticles can enter cells with a lower positive charge than rigid carbon nanotubes.

**Figure 4:**
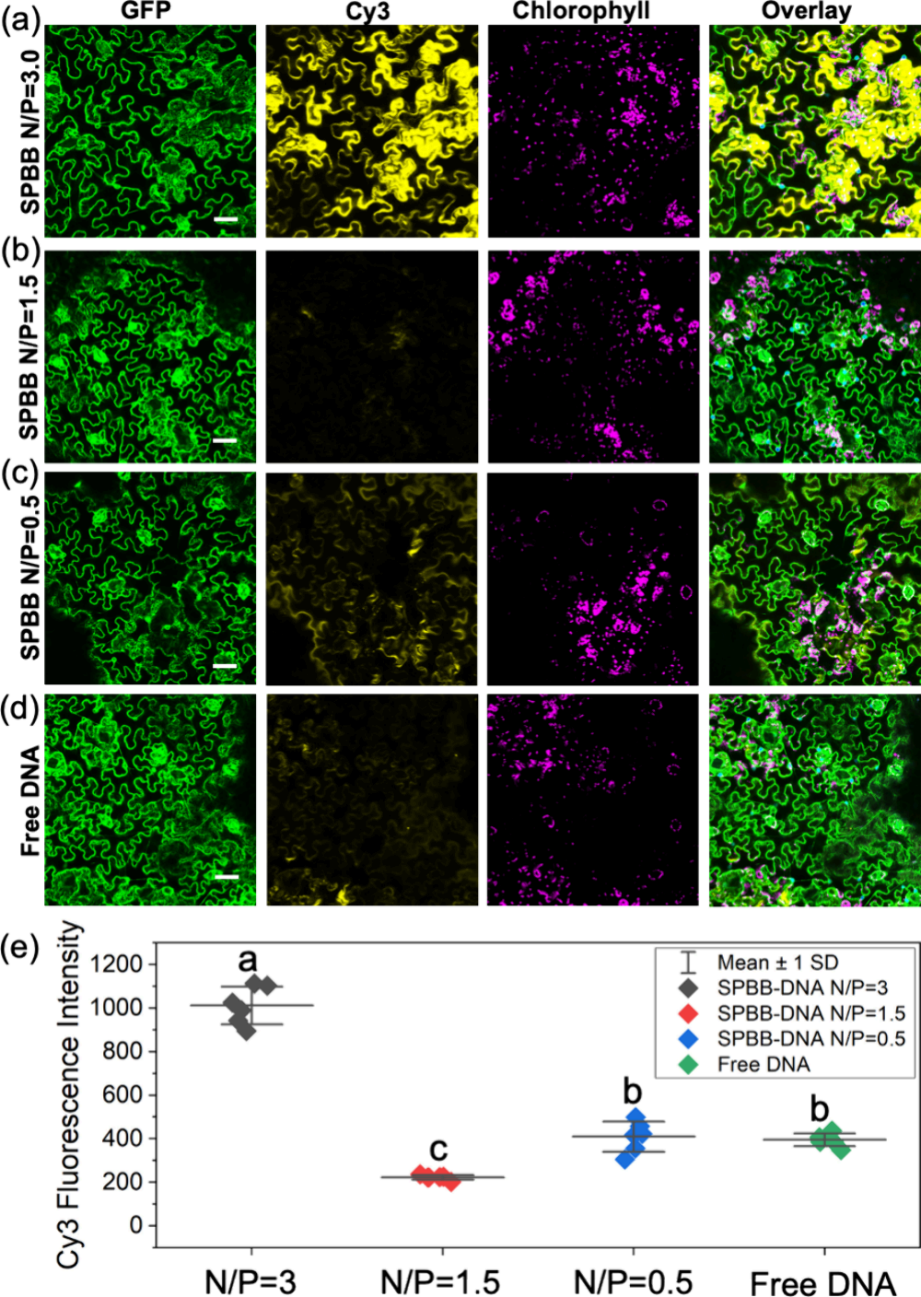
DNA delivery into a transgenic *Nb* plant that constitutively expresses GFP in the cell cytoplasm by SPBB complexed with DNA at different N/P ratios after foliar application. DNA–polymer complex internalization into a plant after foliar application of Cy-3-labeled DNA loaded to SPBB at (a) N/P = 3.0, (b) N/P = 1.5, (c) N/P = 0.5, (d) free DNA. Scale bars, 50 μm. (e) DNA uptake efficiency quantified by Cy3 fluorescence intensity analysis of *Nb* leaf confocal images. Error bars represent standard deviation (*n* = 6). ANOVA testing followed by Fisher’s LSD testing was used for multiple comparisons, *p* ≤ 0.05.

GFP gene expression analysis based on confocal microscopy was implemented in wild-type *Nb* plants to validate and compare the gene delivery efficacy of the different nanocarriers (Figure 5, Figures S8, S9). SP, SPBB, and LPBB were complexed with pDNA at N/P ratios of 0.5, 1.5, and 3.0 and applied to *Nb* plants as described above. GFP expression was assessed 72 h postnoculation. The nuclei of *Nb* plant cells are stained by DAPI to investigate the distribution of expressed GFP. Consistent with the polymer–DNA complex uptake experiments, the GFP signal was only observed for N/P = 3.0 for the SPBB treatment and mainly in the nucleus and along the pavement cell membrane (Figure 5a, Figure S8a). Other N/P ratios and SP, LPBB, and free DNA treatments did not induce detectable GFP expression in *Nb* pavement cells (Figure 5b,c,d, and Figure S9). The GFP expression level was quantified by their fluorescence intensity in plant cells. An N/P of 3.0 had the highest fluorescent intensity of 970.3 ± 75.3 (Figure 5e, Figure S8). Fluorescent intensity weakened for N/P 0.5 and 1.5 treatments, at 815.9 ± 32.9 and 816.2 ± 46.5, respectively, similar to that of free DNA treatment at 775.3 ± 43.8.

**Figure 5:**
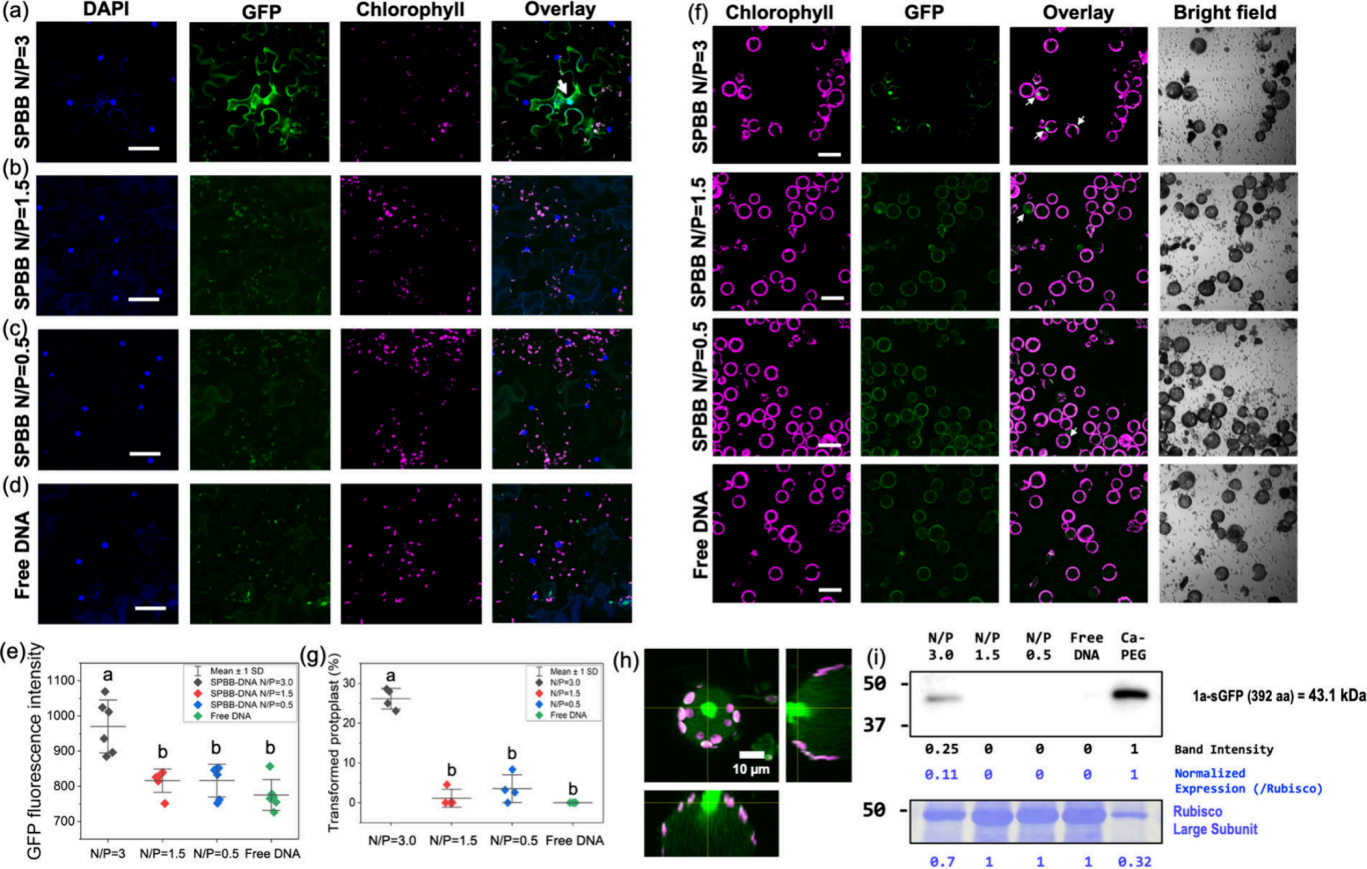
GFP expression in wild-type *Nb* plants enabled by the bottlebrush polymer–DNA complex. Leaves of wild-type *Nb* plants treated with foliar-applied SPBB–DNA at (a) N/P = 3.0, (b) N/P = 1.5, (c) N/P = 0.5, and (d) free DNA by confocal fluorescent microscope 3 days after treatments to determine GFP expression levels. White arrows point to the cells with GFP expression, as the GFP expressed from *UBQ10-Dof1-sGFP(S65T)-NOS* plasmid DNA that has a nuclear localization DOF1a domain sequence to target sGFP(S65T) to the nucleus in the pavement epidermal cells.^9,49,50^ Scale bars: 50 μm. (e) GFP expression in *Nb* plant cells quantified by fluorescence intensity. Images used for fluorescence intensity quantification are shown in Figure S8. (f) Plasmid DNA delivery and expression mediated by SPBB in isolated plant protoplasts, as determined by confocal microscopy. Scale bars, 50 μm. (g) Percentage of the total isolated protoplasts transformed by SPBB–DNA complexes after 24 h of incubation. (h) Orthogonal projections from z-stacks of confocal microscopy images indicating GFP expression in *Nb* protoplasts transfected by SPBB–DNA. Scale bars, 10 μm. (i) GFP expression in protoplasts analyzed by Western blotting.

Polymer–DNA complex charge and size largely affected their ability to transfect plant cells. Lower N/P ratios do not enable an observable GFP expression by confocal microscopy (Figure 5b,c), revealing that larger widths (Figure 2j) and lower net positive charge (Figure S 2d) of polymer–DNA complexes inhibit their entrance into plant cells and subsequent gene expression. This is consistent with previous studies showing that a positive charge enhances nanoparticle uptake into plant cells after foliar application.^45^ However, this has typically required a charge >30 mV; for example, a PEI-functionalized SWCNT–DNA complex that delivered DNA for protein expression in plant cells also had ∼30 mV net positive charge and low width (∼11 nm).^10^ According to the previously established LEEP model, rigid nanoparticles need to possess a net charge above ∼20 mV to autonomously pass through the plant cell membrane.^44,47^ The soft SPBB nanocarriers have delivered DNA into plant cells for expression with a lower net charge, ζ < 20 mV.^37,48^ Therefore, moderately positive charge and small width are essential for biomolecule delivery and expression in the nucleus of plant cells and other organelles. Despite the ability of SPBB-DNA at N/P = 3.0 to deliver DNA into plant cells, the overall GFP expression in intact mature leaves is relatively weak and was below the detection limit of Western blot analysis (data not shown). It is worth noting that SWCNT-mediated DNA expression in plants also could not produce enough GFP for Western blot analysis.^26^ The high net charge of both DNA and polymer nanocarriers and the high molecular weight of plasmid DNA may limit their detachment from nanocarriers postdelivery into cytoplasm and lower their transcription in the nucleus. The limited DNA release is confirmed by DNA gel electrophoresis, as no free plasmid DNA was detected as the pH increased from 6.0 to 7.5 and 9.0 for SPBB–DNA complexes at N/P ratios of 1.5 and 3.0 (Figure S10). This may decrease the DNA accessibility to the plant cell transcription machinery and limit protein expression.^26^

The ability of SPBB to deliver plasmid DNA and trigger gene expression was also investigated in isolated protoplasts. Significant GFP expression was observed for the SPBB–DNA complex at N/P = 3.0 (Figure 5f), with a protoplast transformation efficiency of 26.2 ± 2.6% (Figure 5g), much higher than other N/P ratios and free DNA treatments. Z-stack analysis of the SPBB–DNA transformed protoplast also shows strong GFP expression in the protoplast (Figure 5h). Western blot analysis was performed to further confirm GFP expression in protoplasts transformed by SPBB–DNA complexes. A ∼43.1 kDa GFP-specific protein band was detected by an anti-GFP antibody for SPBB–DNA treatment at N/P = 3.0 (Figure 5i), further confirming GFP expression in protoplasts mediated by polymer nanocarriers. It is worth noting that the GFP expression enabled by SPBB is still much weaker than the traditional protoplast transformation technique, with only ∼11% efficiency compared to the Ca^2+^-PEG-mediated transfection (Figure 5i), likely due to limited DNA release from SPBB (Figure S10).

Plant genetic engineering methods are needed to tackle critical challenges in sustainability, decarbonization, and climate change adaptation. Despite recent advances in nanotechnology-enabled plant genetic engineering, the effect of nanocarrier charge and aspect ratio on their nucleic acid delivery efficiency is not well understood. Here, we demonstrated the ability of a cationic, high aspect ratio, polymer-based DNA delivery platform to deliver nucleic acids in plants and determined how the shape of polymer–DNA complexes affects DNA delivery efficiency in plants. Only the SPBB–DNA complex at N/P = 3.0 with a small overall width and positive charge provided DNA delivery and expression in plants, suggesting that only a restricted range of nanocarriers’ aspect ratios and charges can enable DNA delivery into plant cells. Despite the ability of SPBB to deliver DNA into plant cells, subsequent DNA expression was not extensive, likely a result of limited DNA release from the nanocarriers. Although we achieved relatively weak DNA expression in plant leaves compared to existing biological plant transformation techniques, the polymer nanocarriers reported in this study have identified soft nanocarrier dimensions and nanocarrier to DNA ratios required for efficient DNA delivery into plant cells. Future research needs to develop nanocarriers with lower alkalinity to weaken their binding to DNA and enhance nucleic acid release once they are delivered into the cytoplasm to improve DNA availability for transcription in the nucleus. Compared to the mechanically stiff SWCNTs, the soft polymer nanocarriers enabled GFP expression that was detectable by Western blot protein assays, which indicates a higher gene expression level in protoplasts.^26^ However, the high mechanical strength of SWCNTs enabled cell wall penetration and DNA delivery in plant leaves without enzyme treatment and fostered more efficient DNA delivery and expression in mature plants compared to the polymer carriers. Compared to the nanocarriers that can deliver DNA to human cells, including liposomes, polymeric micelles, and mesoporous silica nanoparticles, the nanocarriers designed for plants typically have smaller widths (<20 nm) and relatively high net charge (>20 mV), which can penetrate the cell wall matrix formed by pectin, cellulose, and hemicellulose, and translocate across plant lipid membranes either by passively opening temporary pores in the lipid membrane or by endocytosis.^44,51,52^ Although SPBB achieved efficient DNA delivery to the cytoplasm, the current nanocarriers require enzyme pretreatments to partly degrade the cell wall. Future research needs to further improve nanocarrier design to enhance penetration through the cell wall for autonomous cell wall crossing. The current SPBB are also made of synthetic polymers with limited biodegradability and are potentially difficult to scale up. Future studies can leverage the nanocarrier design parameters determined in this study for the fabrication of more scalable, biodegradable, and sustainable biopolymer nanocarriers for biomacromolecule delivery to grow new plant cultivars that can enable food, energy, and functional material production at scale.

## Supporting Information Available

Experimental details on materials preparation, characterization, and application to plants, gel permeation chromatography trace of star and bottlebrush polymers, theoretical molecular weight, dispersity, and number-average molecular weight of polymer nanocarriers measured by GPC-MALLS, electrophoretic mobility, apparent zeta potential, and number-average hydrodynamic diameter of polymer nanocarriers before and after complexation with DNA measured by DLS, GFP expression in protoplast extracted from wild-type *Nb* plants 24 h after Ca^2+^/PEG transfection, height profile of free plasmid DNA acquired by AFM, DNA loading into nanocarriers at N/P=3.0 assessed by gel electrophoresis, images used to quantify GFP fluorescence intensity for the SPBB–DNA-treated plants, GFP expression in *Nb* plants after polymer–DNA complex treatment at N/P 0.5 and 1.5, DNA release from SPBB at different pH quantified by agarose gel electrophoresis (PDF)
