# Supplementary material for: High Aspect Ratio Polymer Nanocarriers for Gene Delivery and Expression in Plants
Source: Nano Lett. 2024 Dec 31;25(2):681–90. doi: 10.1021/acs.nanolett.4c04704 (PMC11741140; doi:10.1021/acs.nanolett.4c04704)
Supplement: Supplementary file 1 — nl4c04704_si_001.pdf [file nl4c04704_si_001.pdf]

Supporting information for:

High Aspect Ratio Polymer Nanocarriers for Gene Delivery and Expression in Plants

Yilin Zhang [a,b], Jinwoo Shin [g], Hui Sun [h], Hsin-Fang Chang [h], Michael R. Martinez [c], Lydia A. Perkins [f], Jiajun Yan [c], Yunteng Cao [h], Hairong Wang [a], Juan Pablo Giraldo [i], Krzysztof Matyjaszewski [c], Jen Sheen [g], Robert D. Tilton [b,d,e], Benedetto Marelli [h*], and Gregory V. Lowry [a,b*]*

a. Department of Civil and Environmental Engineering, b. Center for Environmental Implications of Nano Technology (CEINT), c. Department of Chemistry, d. Department of Chemical Engineering, e. Department of Biomedical Engineering, f. Department of Biological Sciences, Carnegie Mellon University, Pittsburgh, Pennsylvania 15213, United States.

g. Department of Molecular Biology and Centre for Computational and Integrative Biology, Massachusetts General Hospital, and Department of Genetics, Harvard Medical School, Boston, MA 02114, United States.

h. Department of Civil and Environmental Engineering, Massachusetts Institute of Technology, Cambridge, Massachusetts 02139, United States.

i. Department of Botany and Plant Sciences, University of California, Riverside, California 92521, United States.

[*] Corresponding authors

Phone: (412) 268-2948; fax: (412) 268-7813; email: glowry@cmu.edu (G.V. Lowry);

Phone: (412) 268-1159; fax: (412) 268-7139; email: tilton@cmu.edu (R.D. Tilton);

Phone: (617) 253-7113; email: bmarelli@mit.edu (B. Marelli);

Experimental section

Materials

β-cyclodextrin (β-CD), 2-(dimethyl amino)ethyl methacrylate (DMAEMA, 98%), 2-bromoisobutyryl bromide (BiBB, 98%), 1,1,4,7,10,10-Hexamethyltriethylenetetramine (HMTETA, 97%), 1-methyl-2-pyrrolidone (NMP), ethyl 2-bromoisobutyrate (EBiB, 98%), copper(I) chloride (CuCl, ≥99.95%), copper(II) chloride ($CuCl_2$, ≥99.95%), potassium fluoride (KF, 99%), basic alumina and chloroform-d ($CDCl_3$) were obtained from Sigma Aldrich. The (2-trimetylsiloxy) ethyl methacrylate (HEMA-TMS) was ordered from Scientific Polymer Products. The anisole (99%) was purchased from Alfa Aesar. Dialysis bags with 8000 Da molecular weight cutoff was purchased from Spectrum lab (Spectra/Por 7). The Label IT Cy3 nucleic acid labeling kit was obtained from Mirus Bio (Madison, WI, U.S.A.). GFP-encoding plasmid UBQ10-Dof1-sGFP(S65)-NOS was obtained from Sheen lab at Harvard medical school.[1] The DMAEMA monomer was purified by passing through basic alumina to remove inhibitor. Other chemicals were used as received without further purification.

Synthesis of 21-armed *β*-CD-*g*-PDMAEMA$_{50}$ star polymers (SP)

β-Cyclodextrin was functionalized by 2-bromoisobutyryl bromide (BiBB) to synthesize *β*-CD-21Br initiator according to previous study. [2–4] The PDMAEMA arms of star polymers were prepared by normal ATRP. Briefly, 0.05 g (1 equiv) of *β*-CD-21Br initiator, 10.4 mL (5250 equiv) DMAEMA, 6.6 mg (4.2 equiv) $CuCl_2$, 0.067 mL (21 equiv) HMTETA and 20.7 mL anisole were added to a sealed Schlenk flask equipped with a stir bar. The Schlenk flask was degassed by purging with N_2 for 40 min and the reaction was frozen by plunging it into liquid nitrogen. The flask was opened briefly to add 24.4 mg (21 equiv) of CuCl powder. The flask was sealed again and purged with N_2 for 30 min before being allowed to warm to room temperature. The reaction was stopped at 20% conversion to yield 21 armed PDMAEMA star polymer (SP) with 50 DMAEMA units in each arm. The products were purified by dialysis (MWCO=8000 Da) against methanol for 4 cycles. The molecular weight distribution of PDMAEMA star polymer was measured by GPC-MALLS (**Figure S1a**).

Synthesis of P(BiBEM-*g*-PDMAEMA$_{50}$)$_{320}$ polymer bottlebrushes (SPBB)

$PBiBEM_{320}$ and $PBiBEM_{1600}$ polymer backbones are made according to previous protocol.[5] The PDMAEMA side chains of bottlebrush were grafted from PBiBEM by normal ATRP. Briefly, 0.05 g (1 equiv) of $PBiBEM_{320}$, 11.96 mL (160000 equiv) of DMAEMA, 3.8 mg (64 equiv) of $CuCl_2$, 0.039 mL (320 equiv) of HMTETA and 23.9 mL anisole were added to Schlenk flask. The Schlenk flask was degassed and frozen to add 14.1 mg (320 equiv) of CuCl. The reaction was stopped at 10% conversion to yield P(BiBEM-*g*-$PDMAEMA_{50})_{320}$ short polymer bottlebrush (SPBB). The product was dialyzed against methanol for 3 cycles (MWCO=8000 Da) and the molecular weight of polymer bottlebrushes was characterized with GPC-MALLS (**Figure S1b**).

Synthesis of P(BiBEM-*g*-$PDMAEMA_{50})_{1600}$ polymer bottlebrushes (LPBB)

Briefly, 0.04 g (1 equiv) of $PBiBEM_{1600}$, 10.98 mL (800000 equiv) of DMAEMA, 3.5 mg (320 equiv) of $CuCl_2$, 0.035 mL (1600 equiv) of HMTETA and 22 mL of anisole were mixed in a sealed Schlenk flask equipped with a stir bar. The reaction was degassed by N_2 and was frozen in liquid nitrogen. 0.013 g (1600 equiv) of CuCl was added to the reaction and the reaction was degassed again by N_2 purging. Reaction was stopped at 10% conversion to yield P(BiBEM-*g*-$PDMAEMA_{50})_{1600}$ long polymer bottlebrush (LPBB).

Polymer characterization by atomic force microscopy, dynamic light scattering and gel permeation chromatography.

Atomic force micrographs were obtained using a Cypher VRS AFM (Asylum Research). All samples were diluted to 5 mg L^{-1} for AFM imaging, to have an optimal density of features on the substrate. In a typical experiment, a 10 μL aliquot of diluted sample was dropped on freshly cleaved mica surface (ϕ = 10 mm, Ted Pella) and air dried before imaging. Images were acquired by tapping mode in air, at a scan rate of 4 - 8 Hz and a resolution of 256 × 256 pixels per image, using FS1500AuD (Asylum Research) probes.[6]

Hydrodynamic diameter, electrophoretic mobility, and apparent zeta potential of polymer nanocarriers were measured in 20 mM MES buffer at pH 5.7 using Malvern Zetasizer nano zs. Apparent zeta potentials (ζ) were calculated from the mobility via the Smoluchowski model. It is important to note that DLS is not an ideal measurement for the hydrodynamic diameter of high aspect ratio materials, but these measurements do indicate that the materials are not aggregating

significantly in 10 mM NaCl solution at pH=5.7. Instead, the AFM height profile better reflects the shape and morphology of polymer nanocarriers with different aspect ratios (**Figure 1b-d**).

Gel permeation chromatography (GPC) was measured via a PSS GPC system with an Agilent 1260 Infinity II isocratic pump, a column set containing 3 PSS GRAM analytical columns (pore sizes: 3000 Å, 3000 Å, 100 Å), an Agilent 1260 Infinity II refractive index (RI) detector, and a PSS SLD2020 multi-angle light scattering (MALS) detector. The column set was kept in a column oven thermostatted at 50 ºC. DMF containing 0.05 M LiBr was used as the eluent. Analysis of polymer signals: PSS WinGPC software (build 9666) was used for molecular weight analysis. RI detector was used as the concentration detector. MALS-based molecular weights were calculated using "concentration × dn/dc" method. The dn/dc value of 0.056 was obtained from previous study.[7] Molecular weight of LPBB (>10M Da) is too high to be separated by the column set used, therefore cannot be accurately measured. The molecular weight of star and bottlebrush nanocarriers varied from 1.9×10^5 to 1.3×10^7 g mol^{-1} with relatively low dispersity (Đ ⩽ 1.20) as measured by gel permeation chromatography with multiangle laser light scattering detector (GPC-MALLS) (**Figure S1** and **Table S1**).

Plant growth

The wild type or GFP overexpressing *Nicotiana benthamiana* (*Nb*) plants used in this study were germinated for 7 days in SunGro Sunshine LC1 Grower soil mix on soil surface. The germinated *Nb* plants were grown at 22 °C with a 16 h light and 8 h dark cycle for 5 weeks before transfection by polymer-DNA complex.

DNA loading into polymer nanocarriers and foliar application.

The plasmid DNA were complexed with polymer nanocarriers by electrostatic interaction. 100 μg plasmid DNA was dissolved in 100 μL of autoclaved MilliQ water to get 1g L^{-1} DNA solution. 15 mg of SP, SPBB or LPBB was dissolved in 3 mL of 20 mM MES buffer at pH 5.7 in an ice bath with sonication (iSonic P4800) to prepare 5 g L^{-1} polymer solution. For all three nanocarriers, 20 μL DNA was mixed with 0.96 μL polymer solution and 29.04 μL MES buffer to prepare polymer-DNA complex with N/P=0.5. 20 μL DNA was mixed with 2.88 μL polymer solution and 27.12 μL MES buffer to get N/P=1.5. 20 μL DNA was mixed with 5.76 μL polymer solution and 24.24 μL MES buffer to get N/P=3. The mixtures were incubated on a vortex mixer

for 30 min and diluted to 1 mL with MES buffer before applied 0.5 mL to each plant leaves. For the N/P=0.5 treatments, where not all plasmid DNA was loaded onto the nanocarriers, a mixture of the polymer-DNA complex and free DNA was infiltrated into the leaves to maintain a consistent DNA mass across different treatments. The Cy3 labeled $(AT)_{15}$ DNA was ordered from IDT and loaded to nanocarriers using the same protocol. [8]

DNA uptake and plant transfection

The youngest mature leaf of 5-week-old *Nb* plants were used for DNA uptake or transfection study. The plants were pretreated with dilute cellulase and macerozyme solution to improve permeability of cell wall.[1] Briefly, 15 mg of cellulase R-10 and 4 mg of macerozyme R-10 were dissolved in a buffer containing 20 mM KCl, 20 mM MES and 10 mM $CaCl_2$. After the enzymes were completely dissolved, the solution was heated at 55 °C for 5 min. After heating, the enzyme solution was filtered using a 0.45 μm syringe filter and diluted 50 times in 20 mM KCl, 20 mM MES and 10 mM $CaCl_2$ before foliar infiltration to the abaxial side of *Nb* leaf with a 1 ml needleless syringe by applying gentle pressure. The polymer-DNA complexes were applied to *Nb* leaves 24h after pretreatment by infiltration with a needle less syringe.[9,10] Each treatment had 3 replicate plants. The treated leaves were imaged by an Olympus FV1200 confocal laser scanning microscope 24 h post treatment for the Cy3 DNA uptake study and 72 h post exposure to quantify GFP expression. The excitation wavelengths were 488 nm for GFP, 550 nm for Cy3 and 633 nm for chlorophyll. The emission wavelengths were 500-520 nm for GFP, 560-580 nm for Cy3 and 700-790 nm for chlorophyll. All images were acquired at ×30 magnification. Three biological replicates were performed with total of 6 images collected for each treatment to quantify Cy3 fluorescence intensity. Each image was analyzed with custom ImageJ analysis to quantify the Cy3 fluorescence intensity value and the intensity values were averaged to calculate a mean fluorescence intensity for each treatment.

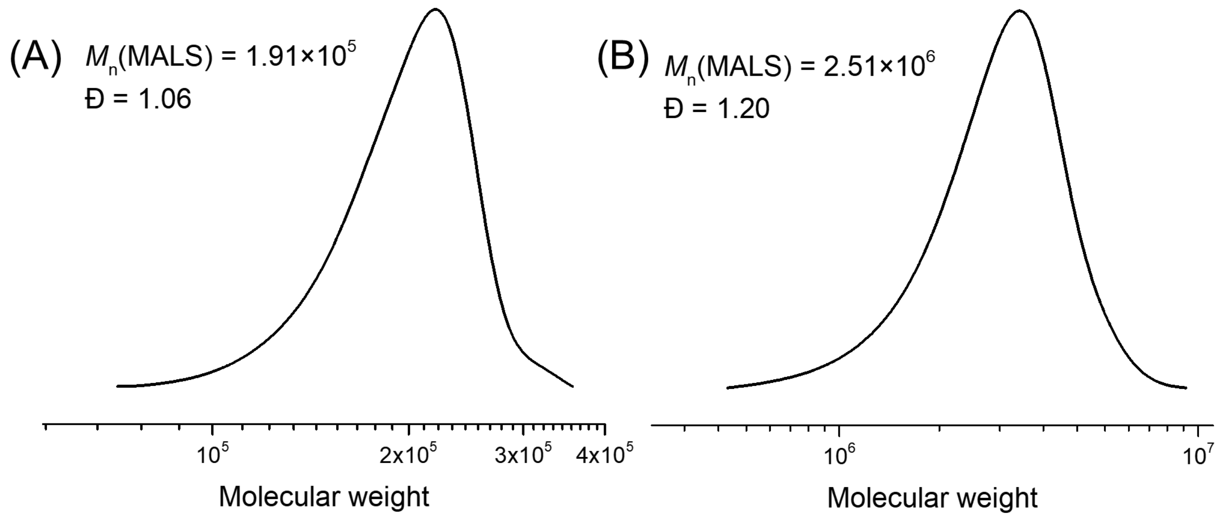

Figure S1. GPC traces of the polymer product of (A) 21-armed $PDMAEMA_{50}$ star polymer (SP) and (B) $P[BiBEM\text{-}g\text{-}PDMAEMA_{50}]_{320}$ bottlebrush (SPBB) in DMF.

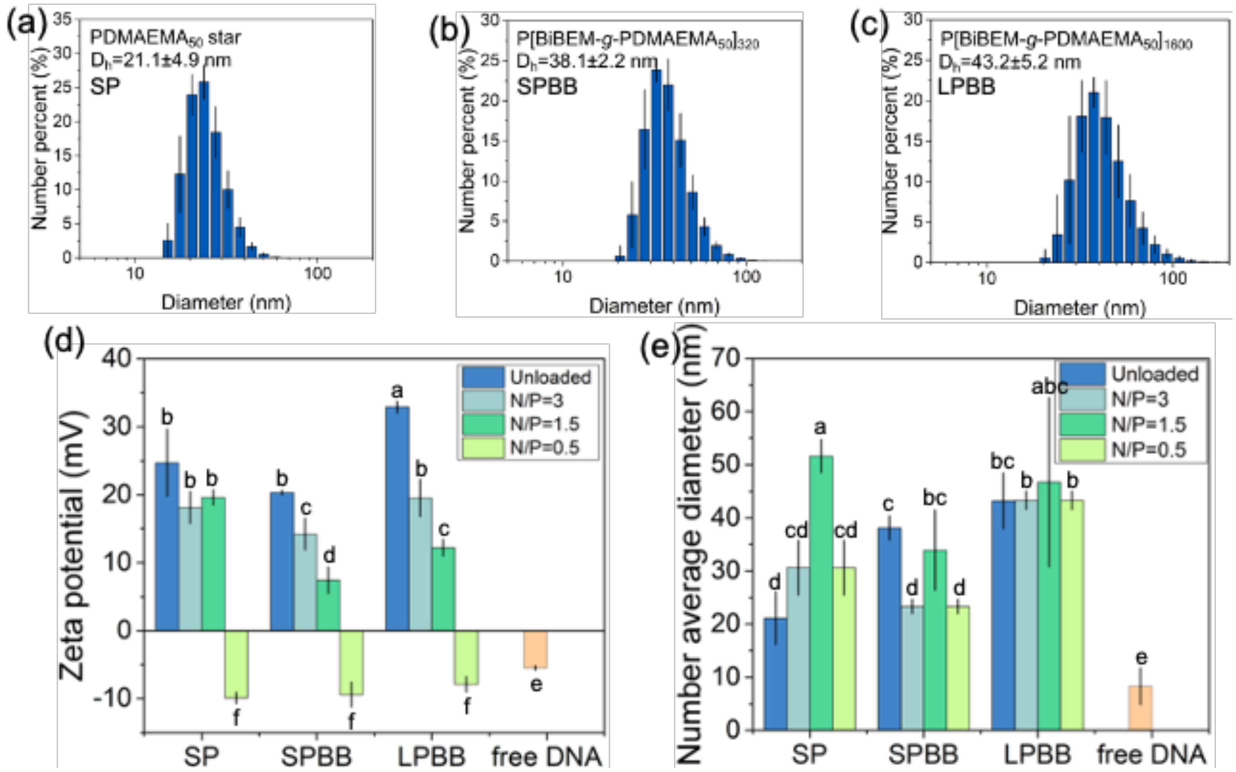

Figure S2. Hydrodynamic diameter of (a) SP (b) SPBB and (c) LPBB at 100 mg L^{-1} polymer concentration in 10 mM NaCl at pH 6.5. (d) Apparent zeta potential of unloaded and pDNA loaded SP, SPBB, LPBB nanocarriers and free DNA at different N to P ratios in 20 mM MES buffer at pH 5.7. Apparent zeta potential was calculated from electrophoretic mobility using the Smoluchowski approximation. (e) Number average hydrodynamic diameter of unloaded and pDNA loaded SP, SPBB, LPBB nanocarriers at different N to P ratios and free DNA in 20 mM MES buffer at pH 5.7. Error bars represent standard deviation (n=3). ANOVA testing followed by Fisher's LSD testing was used for multiple comparisons, $P \leq 0.05$. Dynamic light scattering (DLS) analysis indicated that spherical SP have a hydrodynamic diameter of 21.1±4.9 nm, whereas short rod SPBB and longer worm-like LPBB have similar hydrodynamic diameter at 38.1±2.2 nm and 43.2±5.2 nm, respectively. The hydrodynamic diameter of SP is smaller than SPBB and LPBB ($p < 0.05$), whereas the SPBB and LPBB exhibit similar hydrodynamic diameters.

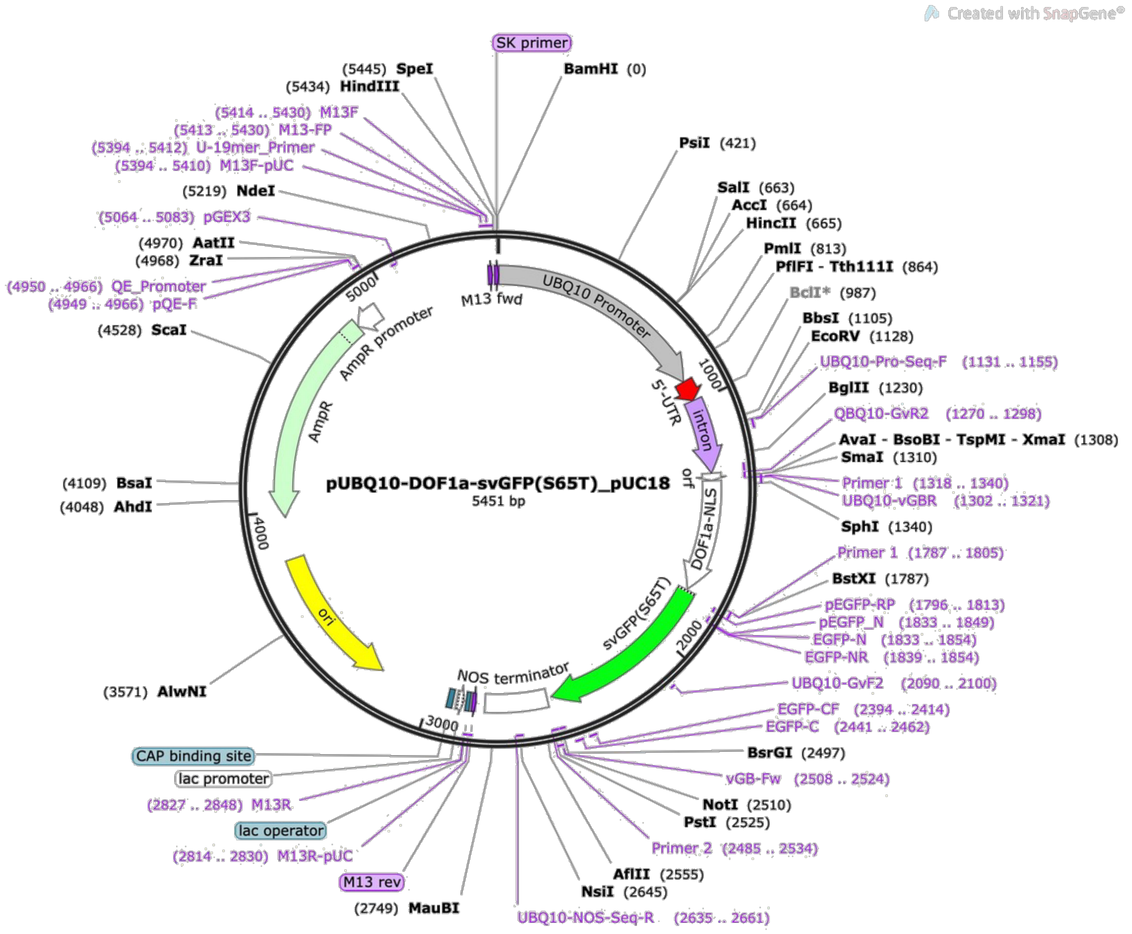

Figure S3. Plasmid map for *UBQ10-DOF1a-sGFP(S65T)-NOS* pDNA encoding GFP that contains a nuclear localization domain (DOF1a) for GFP. [11–13]

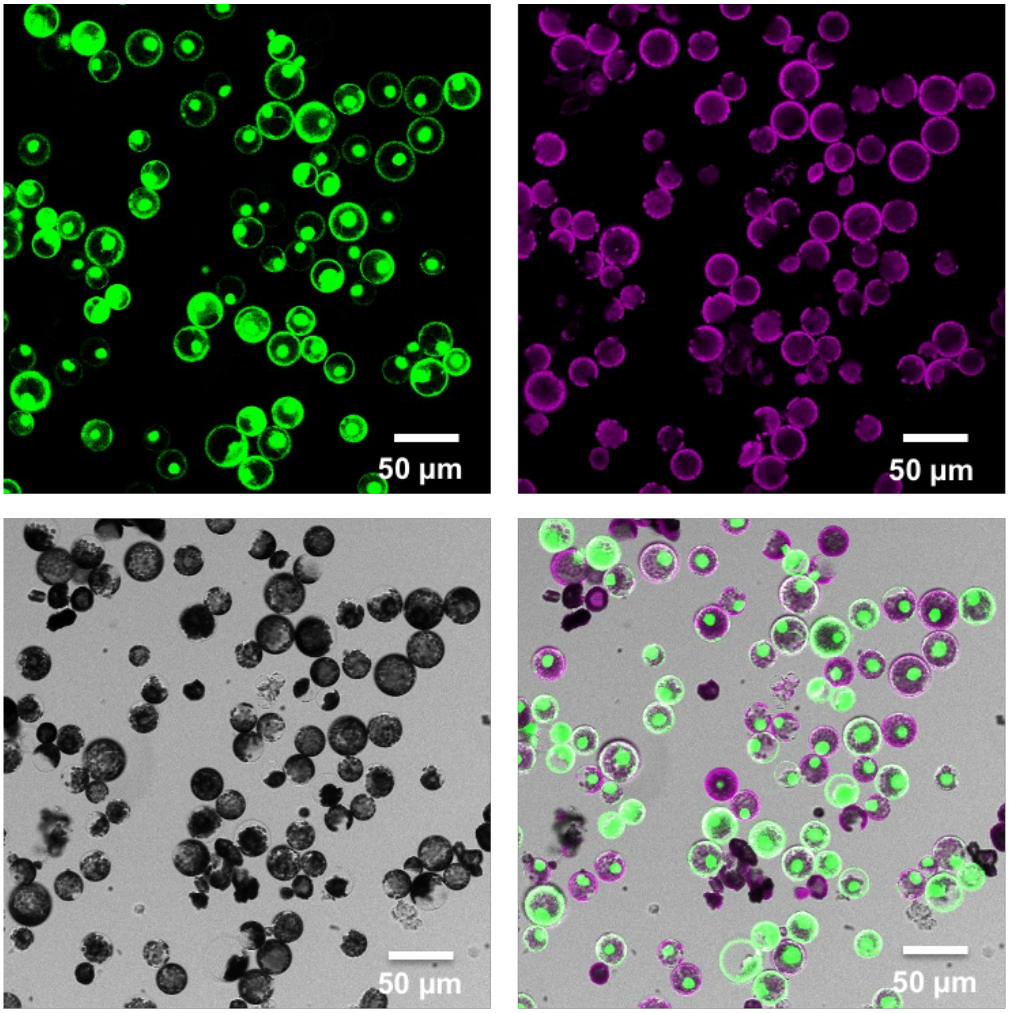

Figure S4. GFP expression in protoplast extracted from wild type *Nb* plants 24 h after Ca^{2+}/PEG transfection with the *UBQ10-DOF1a-sGFP(S65T)-NOS* plasmid DNA imaged by confocal fluorescence microscopy. Note that this plasmid expresses synthetic GFP(S65T) with the DOF1a domain targeting the nucleus.

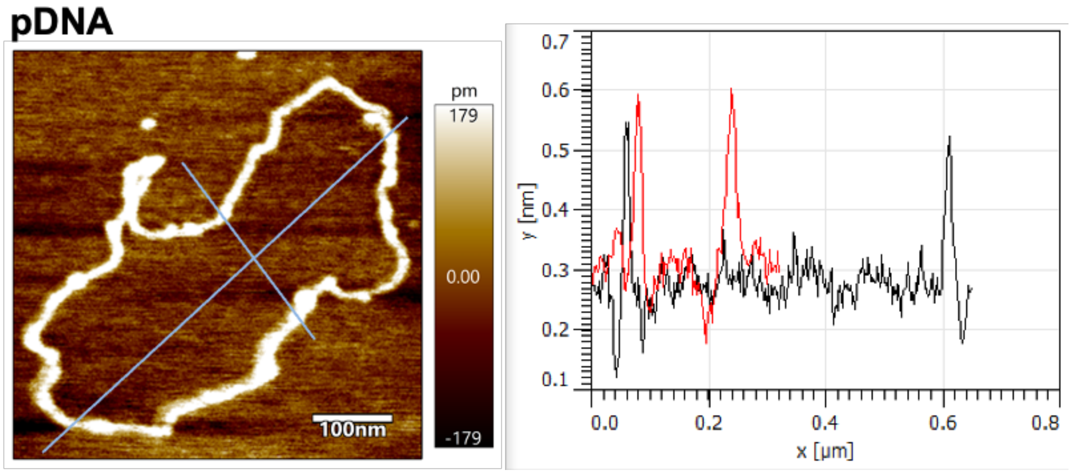

Figure S5. (a) Height profile of free plasmid DNA acquired by atomic force microscopy. (b) AFM width of SP, SPBB, and LPBB complexed with plasmid DNA at N/P=3.0. (c) AFM width of unloaded SPBB and SPBB complexed with plasmid DNA at N/P=0.5, 1.5, and 3.0. Error bars represent standard deviation (n=5). ANOVA testing followed by Fisher's LSD testing was used for multiple comparisons, P≤ 0.05.

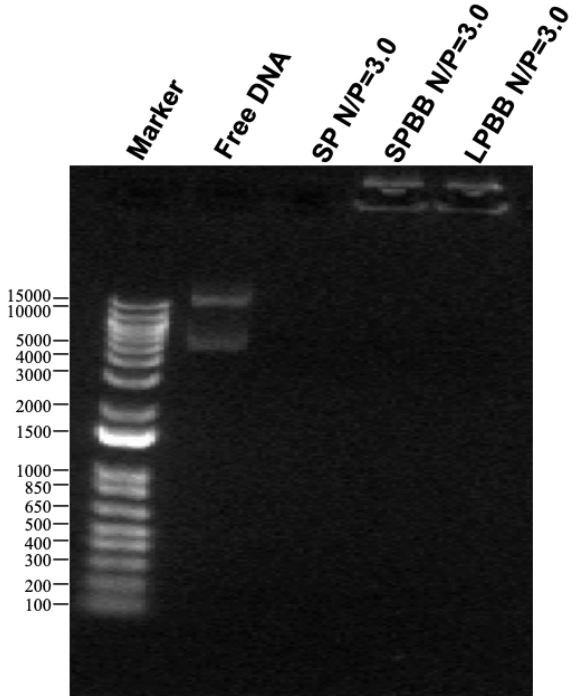

Figure S6. DNA loading into SP, SPBB and LPBB quantified by agarose gel electrophoresis of plasmid DNA complexed at 3.0 N/P ratios.

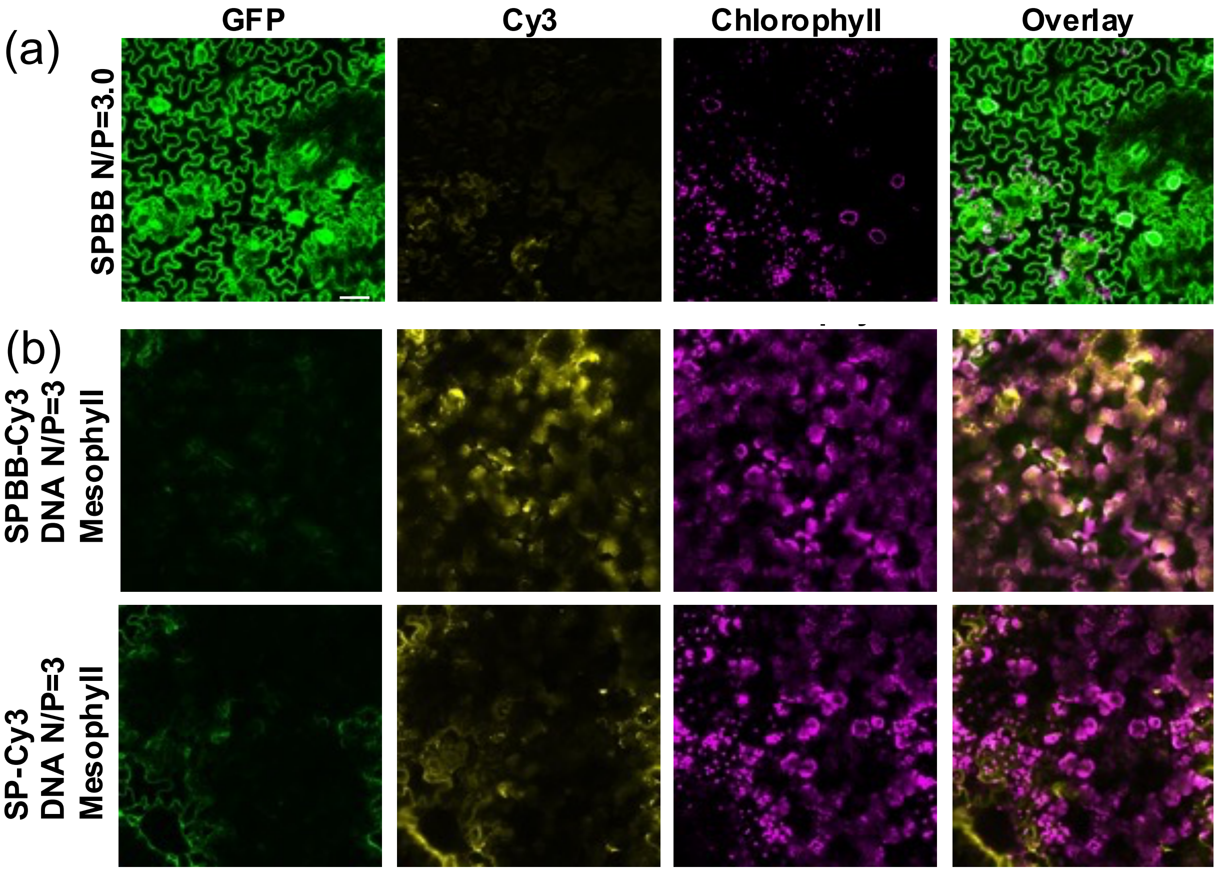

Figure S7. DNA delivery into a transgenic Nb plant that constitutively expresses GFP in the cell cytoplasm (a) by SPBB at N/P=3.0 without enzyme pretreatment. Limited delivery was observed suggesting that the cell wall was significant barrier to uptake of the polymer-pDNA complexes. (b) by SPBB and SP at N/P=3.0 with enzyme pretreatments. Scale bars, 50 μm.

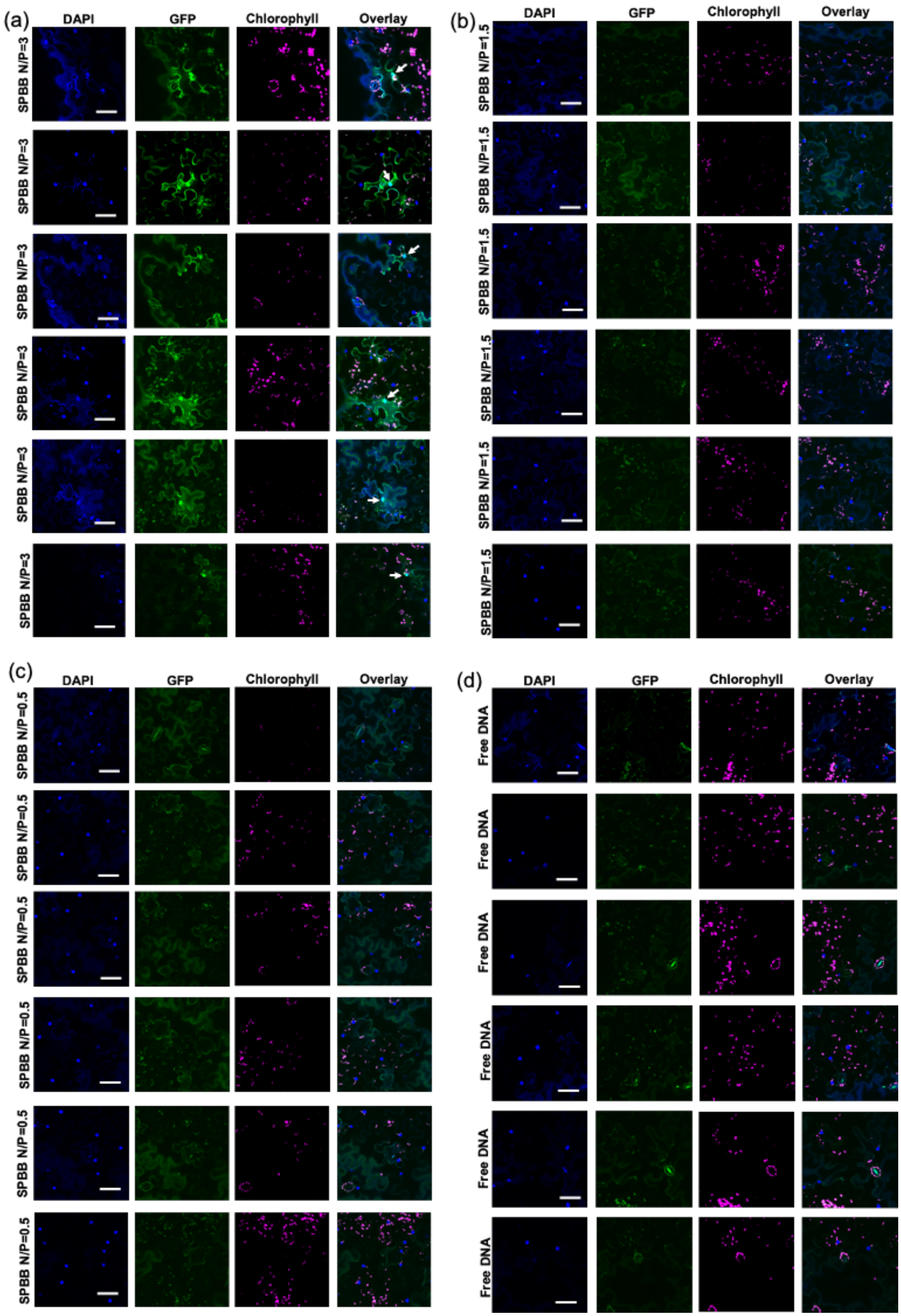

Figure S8. GFP expression in *Nb* plants enabled by bottlebrush polymer-DNA complex. Leaf cell of wild type Nb plants treated with foliar applied SPBB-DNA at (a) N/P=3.0, (b) N/P=1.5,

(c) N/P=0.5, and (d) free DNA. Green: GFP, blue: DAPI, magenta: chlorophyll. Scale bars, 50 μm.

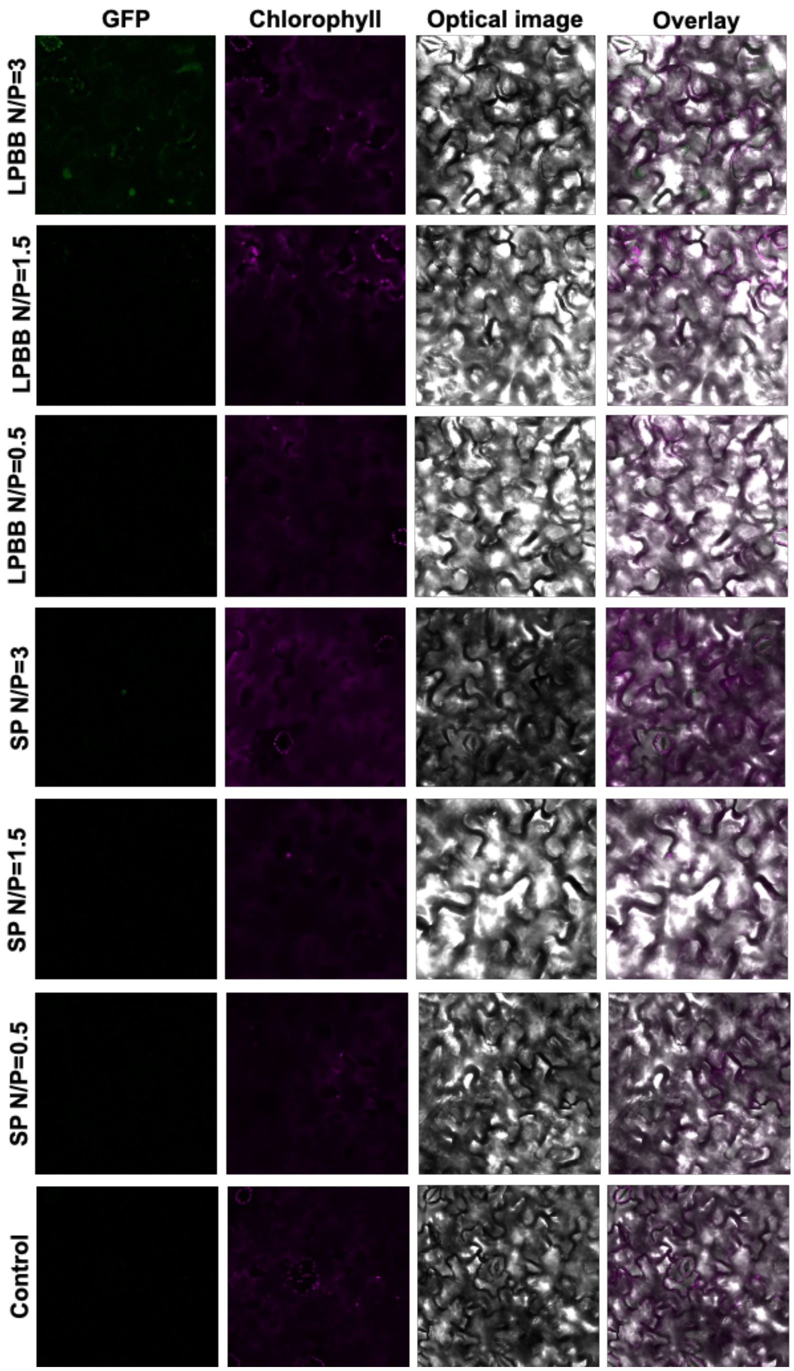

Figure S9. GFP expression in *Nb* plants after polymer-DNA complex treatment. Leaf cell of wild type *Nb* plants treated with foliar applied SP and LPBB at N/P=0.5, 1.5, and 3.0 in 20 mM MES buffer at pH 5.7 and untreated control plant imaged by confocal fluorescent microscope 3 days after treatments. No significant GFP expression is observed from these treatments.

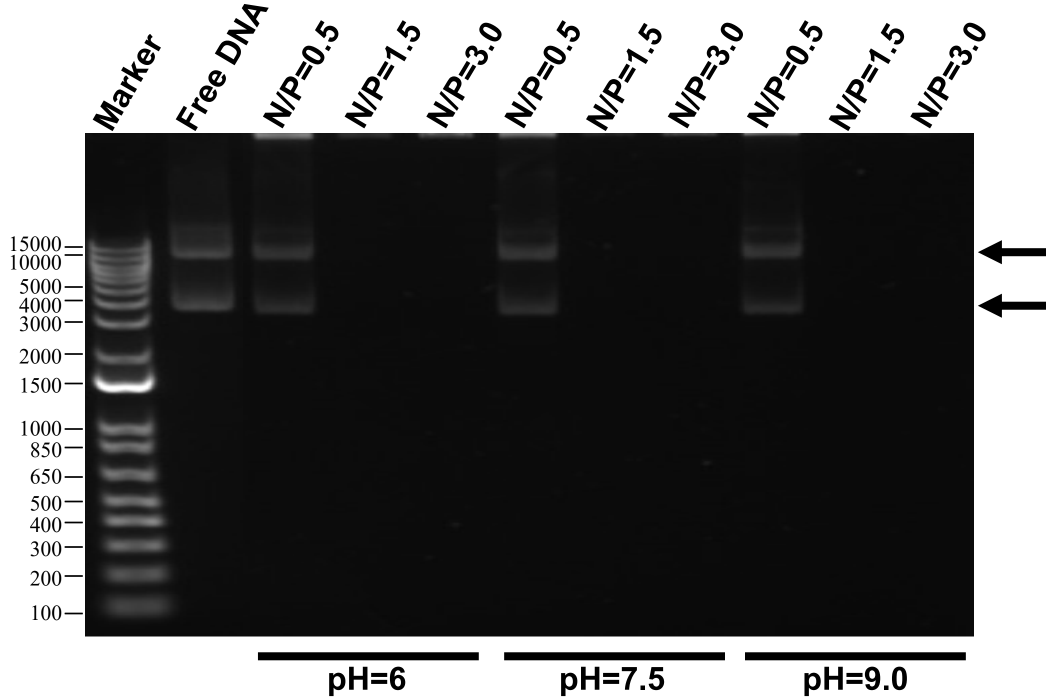

Figure S10. DNA release from SPBB at different pH quantified by agarose gel electrophoresis of pDNA complexed to SPBB at 0.5, 1.5, and 3.0 N/P ratios and incubated in 50 mM phosphate buffer at pH 6.0, 7.5 and 9.0 for 1h. M: DNA ladder. Black arrows indicate supercoiled (below) and circular (upper) pDNA bands.

Table S1. Theoretical number average molecular weights (M_n) and M_n of PDMAEMA star polymers and P[BiBEM-*g*-PDMAEMA] polymer bottlebrushes in DMF measured by GPC-MALLS. Molecular weight of P[BiBEM-g-$PDMAEMA_{50}]_{320}$ bottlebrush (LPBB) is higher than the detection range of GPC-MALLS, thus cannot be measured.

Sample	$M_{n,\ theory}$	$M_{n,\ MALS}$	*Đ*
**$PDMAEMA_{50}$ star polymer**	1.7×10^5	1.91×10^5	1.20
**P[BiBEM-*g*-$PDMAEMA_{50}]_{320}$ bottlebrush**	2.6×10^6	2.51×10^6	1.05
**P[BiBEM-*g*-$PDMAEMA_{50}]_{1600}$ bottlebrush**	1.3×10^7	/	/

Table S2. Electrophoretic mobility, apparent zeta potential and number average hydrodynamic diameter of polymer nanocarriers before and after complexation with DNA.

**Sample**		**Electrophoretic mobility (μm cm V^{-1} s^{-1})[a]**	**z (mV)[b]**	**D_h[c](nm)**
**$PDMAEMA_{50}$ star (SP)**		1.93±0.39	24.7±4.9	21.1±4.9
**P[BiBEM-*g*-$PDMAEMA_{50}]_{320}$ bottlebrush (SPBB)**		1.59±0.02	20.3±0.3	38.1±2.2
**P[BiBEM-*g*-$PDMAEMA_{50}]_{1600}$ bottlebrush (LPBB)**		2.58±0.07	32.9±0.8	43.2±5.2
**SP-DNA complex**	N/P=0.5	-0.78±0.06	-9.9±0.8	39.3±6.2
	N/P=1.5	1.54±0.08	19.6±1.1	51.6±3.1
	N/P=3	1.42±0.18	18.1±2.3	30.6±5.1
**SPBB-DNA complex**	N/P=0.5	-0.74±0.14	-9.4±1.8	27.4±4.3
	N/P=1.5	0.58±0.15	7.4±1.9	33.9±7.5
	N/P=3	1.11±0.18	14.2±2.3	23.3±1.3
**LPBB-DNA complex**	N/P=0.5	-0.62±0.08	-7.9±1.1	35.6±9.2
	N/P=1.5	0.95±0.1	12.2±1.2	46.7±15.9
	N/P=3	1.54±0.21	19.5±2.7	43.3±1.7
**Free DNA**		1.13±0.08	-5.5±0.3	8.3±3.4

[a] Electrophoretic mobility of polymers measured in 20 mM MES buffer at pH 5.7 with 100 mg L^{-1} polymer concentration. [b] Zeta potential was converted from electrophoretic mobility based on Smoluchowski equation. [c] Number average hydrodynamic diameter measured by DLS at 100 mg L^{-1} polymer concentration at pH 5.7 in 20 mM MES buffer.

References

(1) Yoo, S.-D.; Cho, Y.-H.; Sheen, J. Arabidopsis Mesophyll Protoplasts: A Versatile Cell System for Transient Gene Expression Analysis. *Nature Protocols* **2007**, 2, 1565–1572.

(2) Zhang, Y.; Fu, L.; Li, S.; Yan, J.; Sun, M.; Pablo Giraldo, J.; Matyjaszewski, K.; D. Tilton, R.; V. Lowry, G. Star Polymer Size, Charge Content, and Hydrophobicity Affect Their Leaf Uptake and Translocation in Plants. *Environ Sci Technol* **2021**, *55* (15), 10758–10768.

(3) Zhang, Y.; Yan, J.; Avellan, A.; Gao, X.; Matyjaszewski, K.; Tilton, R. D.; Lowry, G. V. Temperature- And PH-Responsive Star Polymers as Nanocarriers with Potential for in Vivo Agrochemical Delivery. *ACS Nano* **2020**, *14* (9), 10954–10965.

(4) Zhang, Y.; Fu, L.; Martinez, M. R.; Sun, H.; Nava, V.; Yan, J.; Ristroph, K.; Averick, S. E.; Marelli, B.; Giraldo, J. P.; Matyjaszewski, K.; Tilton, R. D.; Lowry, G. V. Temperature-Responsive Bottlebrush Polymers Deliver a Stress-Regulating Agent in Vivo for Prolonged Plant Heat Stress Mitigation. *ACS Sustain Chem Eng* **2023**, *11* (8), 3346–3358.

(5) Zaborniak, I.; Chmielarz, P.; Martinez, M. R.; Wolski, K.; Wang, Z.; Matyjaszewski, K. Synthesis of High Molecular Weight Poly(n-Butyl Acrylate) Macromolecules via SeATRP: From Polymer Stars to Molecular Bottlebrushes. *Eur Polym J* **2020**, *126*, 109566.

(6) Sun, H.; Marelli, B. Polypeptide Templating for Designer Hierarchical Materials. *Nature Communications 2020 11:1* **2020**, *11* (1), 1–13.

(7) Methacrylate, N.; Situ, I. Antimicrobial Activity of Hybrid Nanomaterials Based on Star and Linear Polymers Of. *Materials* **2020**, *13*, 3037.

(8) Demirer, G. S.; Zhang, H.; Goh, N. S.; Pinals, R. L.; Chang, R.; Landry, M. P. Carbon Nanocarriers Deliver SiRNA to Intact Plant Cells for Efficient Gene Knockdown. *Sci Adv* **2020**, *6* (26).

(9) Thagun, C.; Horii, Y.; Mori, M.; Fujita, S.; Ohtani, M.; Tsuchiya, K.; Kodama, Y.; Odahara, M.; Numata, K. Non-Transgenic Gene Modulation via Spray Delivery of Nucleic Acid/Peptide Complexes into Plant Nuclei and Chloroplasts. *ACS Nano* **2022**, *16*, 3521.

(10) Hu, P.; An, J.; Faulkner, M. M.; Wu, H.; Li, Z.; Tian, X.; Giraldo, J. P. Nanoparticle Charge and Size Control Foliar Delivery Efficiency to Plant Cells and Organelles. *ACS Nano*. **2020**, 7970–7986.

(11) Jiang, W.; Bush, J.; Sheen, J. A Versatile and Efficient Plant Protoplast Platform for Genome Editing by Cas9 RNPs. *Front Genome Ed* **2021**, *3*.

(12) Chiu, W. L.; Niwa, Y.; Zeng, W.; Hirano, T.; Kobayashi, H.; Sheen, J. Engineered GFP as a Vital Reporter in Plants. *Current Biology* **1996**, *6* (3), 325–330.

(13) Yanagisawa, S.; Sheen, J. Involvement of Maize Dof Zinc Finger Proteins in Tissue-Specific and Light-Regulated Gene Expression. *Plant Cell* **1998**, *10* (1), 75–89.
